# Post-ablation Dyspnea a Case Report to Highlight the Differential Diagnoses

**DOI:** 10.7759/cureus.17793

**Published:** 2021-09-07

**Authors:** Amr Salem, Ahmed Salem, Juan Fernando Ortiz, Ronny A Cohen

**Affiliations:** 1 Hospital Medicine, Brown University, Providence, USA; 2 Internal Medicine, Alexandria Faculty of Medicine, Alexandria, EGY; 3 Neurology, Universidad San Francisco de Quito, Quito, ECU; 4 Neurology, Larkin Community Hospital, Miami, USA; 5 Cardiology, Woodhull Hospital, New York University (NYU) School of Medicine, New York, USA

**Keywords:** atrial fibrillation, cardiac ablation, differential diagnosis, dyspnea, pericarditis

## Abstract

The diagnosis of post-cardiac ablation pericarditis is difficult as it requires the exclusion of the more common causes of chest pain, but in the right setting, non-invasive diagnostic tools are adequate. Here we present the case of a 60-year-old man who underwent atrial fibrillation ablation and subsequently developed severe mid-sternal chest pain and dyspnea one day later without significant electrocardiographic findings, a mildly elevated troponin T, and elevation of the right hemidiaphragm. The patient was managed conservatively. A two-dimensional transthoracic echocardiogram showed no regional wall motion abnormalities, significant transvalvular gradients, but showed minimal pericardial effusion. A sniff test was negative for diaphragmatic paralysis. After the diagnosis, the patient’s symptoms resolved with non-steroidal anti-inflammatory drugs and colchicine. This case of pericarditis after cardiac ablation highlights the possible differential diagnosis when confronted with post-ablation cardiac symptoms. Despite the classic presentation, the electrocardiogram showed no significant ST/PR changes. In the right clinical setting, non-invasive imaging may be appropriate management.

## Introduction

Atrial fibrillation (AF) is common, affecting between three and six million Americans, but is expected to increase to six to 12 million affected individuals by 2050 [1]. There are two main goals to the management of AF: first, management of the arrhythmia through a rhythm-versus-rate control strategy, and second, stroke prevention through anticoagulation and/or left atrial appendage isolation [2]. Rhythm management can be achieved through arrhythmia-modifying drugs, cardioversion, or pulmonary vein ablation [2]. Unfortunately, antiarrhythmic medications are limited by side effects in 30% of patients, with 10% needing to stop due to side effects and 14% stop due to inefficacy [3]. Catheter ablation has several advantages over medical therapy, with superior maintenance of sinus rhythm and reduced admission rates and mortality in AF patients with heart failure [3-5]. However, there are no definite benefits to catheter ablation in non-heart failure patients [6], with the catheter ablation vs antiarrhythmic drug therapy in atrial fibrillation (CABNA) trial showing no differences in terms of composite death, stroke, bleeding, or cardiac arrest over a five-year period compared to medical therapy [7]. The European Society of Cardiology (ESC), however, gave a grade 1 recommendation for use of catheter ablation in patients who improved after initial pulmonic vein isolation [8].

Catheter ablation is a complex electrophysiological procedure that involves manipulation and ablation in the delicate atria near vital structures including the esophagus, phrenic nerve, mitral apparatus, and the coronaries [9]. Hence, catheter ablation has a significant risk of complications [10-11], and risk stratification is prudent. Major complications include life-threatening cardiac tamponade, mediastinitis, and permanent injury, particularly stroke and vascular access complications [10]. Pericardial complications include post-ablation pericarditis, pericardial effusion, and Dressler syndrome [9-10,12], with the incidence of post-ablation pericarditis reported to be anywhere between 0 and 50% [9]. Complications are more common in women, extremes of body mass index (BMI), patients with structural heart disease, and repeat procedures [13]. Complications are also more likely in low-volume centers and with inexperienced operators [14-15]. Complications vary according to ablation type; for example, radiofrequency ablation is associated with a higher risk of pericardial effusion and tamponade, cerebrovascular accidents, esophageal injury, and pulmonary vein stenosis, while cryoablation is associated with a higher incidence of phrenic nerve palsy and vagal nerve injury [4,16]. The rate of pericardial disease post-ablation increased between 2009 and 2014, showing a 2.8-fold increase in the incidence of post-ablation pericarditis [17]. This is probably due to an increased referral rate for the procedure.

Patients with hypertrophic obstructive cardiomyopathy (HOCM) may benefit from catheter-based ablation, although this has not been tested in randomized studies. However, ablation is considered a safe and effective approach for the management of AF in these patients. Unfortunately, the success rate is lower than in non-HOCM patients, and repeat ablation procedures are required to achieve sinus rhythm [18]. Here we describe the case of a 60-year-old man who underwent AF ablation and subsequently developed severe mid-sternal chest pain and dyspnea, which was finally diagnosed as post-ablation pericarditis. Presenting this case provides the opportunity to discuss the differential diagnoses that physicians must consider when confronted with post-ablation cardiac symptoms.

## Case presentation

A 60-year-old obese male with a BMI of 40, Hypertension, obstructive sleep apnea (OSA) non-compliant with continuous positive airway pressure (CPAP), and HOCM presented for elective ablation due to treatment-refractory AF and chronic heart failure. He had a past medical history of paroxysmal atrial fibrillation treated by cryoballoon ablation in 2010, HOCM treated with alcohol septal ablation and cryoablation, and paroxysmal ventricular tachycardia managed with a dual-chamber pacemaker; as he was previously deemed high risk for high-grade heart block with septal ablation. After intracardiac thrombus was ruled out by transesophageal echocardiography, the patient underwent radiofrequency ablation with isolation of the right inferior and superior pulmonary veins and the creation of a tricuspid isthmus line. The patient initially received a bolus of 12,000 units of heparin followed by additional heparin as needed to maintain an activated coagulation time (ACT) above 300 seconds. The patient tolerated the procedure well and maintained an ACT > 300 seconds with heparin, with subsequent protamine reversal post-procedure. The patient was discharged the next day on his usual novel anticoagulant rivaroxaban 20 mg daily, verapamil, and disopyramide.

**Figure 1:**
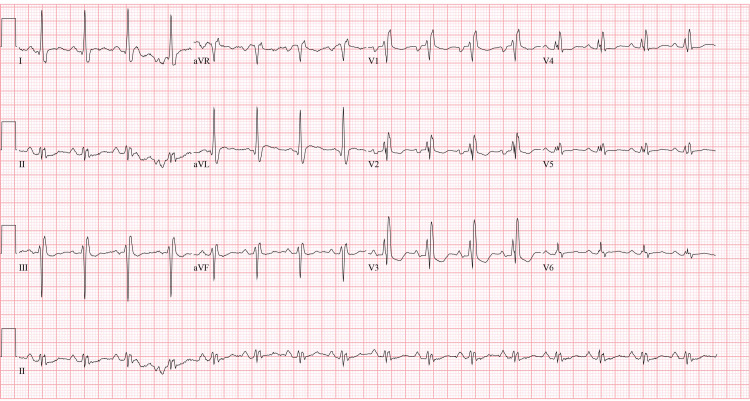
ECG showing normal sinus rhythm, right bundle branch block, nonspecific ST/T-wave abnormalities. ECG: Electrocardiogram

**Figure 2:**
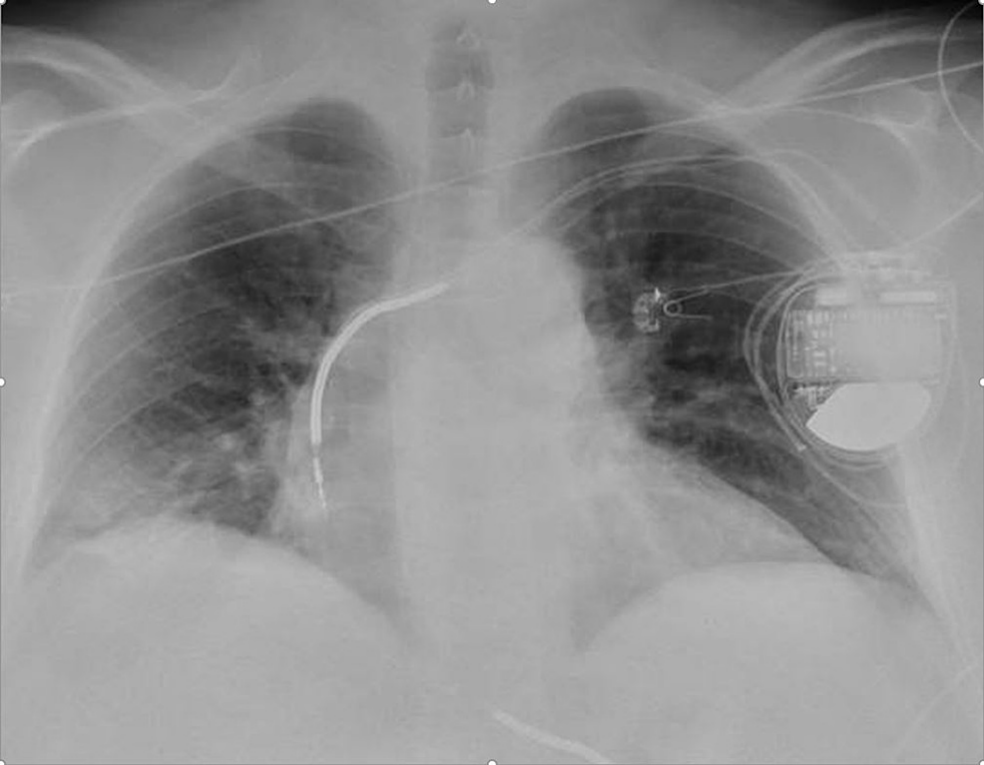
Chest x-ray showing elevated hemidiaphragm, which was confirmed to be chronic after comparison with prior x-rays. Paralysis was also ruled out with a negative sniff test.

The next day, the patient returned with seven out of 10 mid-sternal sharp chest pain. The pain was non-radiating and improved by leaning forwards, worse when lying supine, with no response to nitrates given by emergency medical services (EMS). A review of systems was negative for fever, chills, cough, sick contacts, heartburn, or history of trauma. Vitals showed blood pressure (BP) at 110/80 mmHg, pulse (P) 65 bpm, respiratory rate (RR) 18 breaths per minute, and temperature (T) 98.6 degrees Fahrenheit. On general physical examination, the patient was obese, well developed, and nontoxic. On examination of the head, eyes, ears, nose, and throat (HEENT), pupils were equal and reactive bilaterally, extraocular motility was intact, no jugular venous distension (JVD) was seen. On cardiac examination, no murmur, gallop but with a pericardial rub was present. Chest examination showed scattered wheezing but no chest wall tenderness was present. Electrocardiography showed normal sinus rhythm, nonspecific ST/T wave abnormalities, old right bundle branch block (Figure 1). Troponin T was elevated at 0.52, 0.42, and 0.41 ng/mL, at one, six, and 10 hours from presentation (reference range: 0.006 and 0.06 ng/ml) and he had a hemoglobin of 12.7 g/dL, white blood cell (WBC) of 6.7 k/ul. Inflammatory markers were elevated with a C-reactive protein (CRP) of 8.9 mg/dl and erythrocyte sedimentation rate (ESR) of 17 mm/hr. A two-dimensional transthoracic echocardiogram (TTE) was performed, which showed minimal pericardial effusion, a left ventricular ejection fraction of 60-75%, grade 2 diastolic dysfunction with a pseudo-normal filling pattern, mid-cavitary obliteration without obstruction, and mild systolic anterior motion without evidence of outflow obstruction. A chest x-ray showed elevation of the right hemidiaphragm (Figure 2). A sniff test (diaphragm fluoroscopy) showed no evidence of paralysis, with a collateral history confirming prior phrenic nerve injury related to previous AF ablation; indeed, upon comparison with a previous x-ray, the hemidiaphragm elevation proved to be chronic. The patient was initially started on a heparin-based anticoagulation acute coronary syndrome protocol. This was, however, discontinued, and switched to his home rivaroxaban as the diagnosis of the acute coronary syndrome was highly unlikely due to a combination of a recent negative heart catheterization for coronary artery disease, positional chest pain, and indolent troponin trajectories (flat troponins) atypical for the acute coronary syndrome, along with an absence of significant ECG abnormalities or imaging evidence of acute coronary syndrome. The patient also met the diagnostic prerequisites for pericarditis, i.e. three out of four criteria, where normally two criteria are sufficient. These included 1) Pleuritic sharp chest pain improved by leaning forward (present in 85-90% of cases), 2) pericardial friction rub (present in 1/3 of patients), and 3) pericardial effusion which is present in 60% of patients. The fourth-ECG changes i.e. widespread ST depression or PR elevation-was not evident in our case. The patient was continued on rivaroxaban.

The patient was diagnosed as having suffered post-procedural pericarditis and discharged home, on day two of admission, on colchicine and aspirin 325 mg along with his home verapamil, disopyramide, and rivaroxaban. The decision was made to discharge the patient, as he didn't demonstrate fever and no evidence of large pericardial effusion on transthoracic echocardiogram (TTE). A week later, the patient was seen as an outpatient, his chest pain had nearly resolved, and inflammatory markers had normalized. According to an interrogation of his implantable cardio-defibrillator, the atrial arrhythmia burden was significantly reduced three months later. Compliance with continuous positive airway pressure was encouraged. 

## Discussion

This case highlights the differential diagnosis of postprocedural complications after AF ablation. Our patient had several risk factors for ablation-related complications: obesity (body mass index of 40), a repeat procedure after two failed ablations, and HOCM, a known risk factor for AF recurrence. In our case, the patient had to be readmitted for evaluation and observation [11], which allowed us to diagnose pericarditis based on the typical positional chest pain, pericardial friction rub, and pericardial effusion, with elevated inflammatory biomarkers, also supporting the diagnosis.

In the emergency department, the differential included acute coronary syndrome, arrhythmic complications, diaphragmatic paralysis, stress-induced cardiomyopathy, pericarditis, and pulmonic vein stenosis. Other differentials not directly related to the procedure including Tietz's syndrome, gastroesophageal reflux disease (GERD), chest trauma were considered and excluded as history was not suggestive, also in the presence of a clear temporal relation to the procedure these diagnoses were considered less likely. The patient was diagnosed with pericarditis after the exclusion of the other differential diagnoses. The diagnosis was based on the ESC criteria considering the typical pericardial pain, new pericardial effusion, and pericardial rub. [8] It was also supported by the presence of inflammatory biomarkers. Interpretation of the ECG in our case is likely limited by baseline conduction abnormalities. It is noteworthy, however, that the typical ECG features for pericarditis are only present 60% of the time. [8] The usual diagnosis of myocardial infarction (MI) cannot be applied in the case of ablation as biomarkers (creatine kinase and troponin) are consistently elevated [11]. However, MI could be excluded in this case based on the lack of significant ST/T wave abnormalities, new pathological Q waves on ECG, together with an absence of regional wall motion abnormalities on echocardiography [11].

Phrenic nerve paralysis was included in the differential diagnosis for this patient as he had an elevated right hemidiaphragm. However, this was ruled out by a negative sniff test. In addition, pulmonary vein stenosis was also considered; however, this usually occurs two to five months after the procedure [19-21]. Finally, cardiac tamponade, a life-threatening complication, has been reported in about 1% of radiofrequency ablation procedures [22], but there was no hemodynamic or echocardiographic evidence of this life-threatening complication in our case.

Although the rate of post-ablation complications has steadily decreased, all physicians should be aware of the potential complications of this procedure, including post-ablation pericarditis. In addition, the rate of post-ablation pericarditis has almost tripled, contributing to increased utilization of healthcare resources [17], most likely related to an increased number of referrals for such procedures. Improved diagnostic tools could have also contributed.

In this case, the diagnosis was difficult as, despite the typically positional chest pain, there were no significant ECG changes, possibly due to the baseline conduction abnormalities and known HOCM. Nevertheless, the diagnosis was adequately made based on clinical criteria (chest pain, friction rub, and pericardial effusion) and a lack of echocardiographic findings suggesting other diagnoses. Unnecessary invasive procedures like heart catheterization were hence avoided.

## Conclusions

Pericarditis should always remain in the differential diagnosis of post-ablation complications, thereby ensuring its rapid diagnosis while avoiding invasive procedures unless indicated. HOCM represents a risk factor for post-ablation complications. Despite the decreased rate of complications, the incidence of post-ablation pericarditis is on the rise. 
